# Comparative Transcriptome Analysis of Cold Tolerance Mechanism in Honeybees (*Apis mellifera sinisxinyuan*)

**DOI:** 10.3390/insects15100790

**Published:** 2024-10-11

**Authors:** Jinqiong Shan, Ruiyi Cheng, Tuohudasheng Magaoya, Yujie Duan, Chao Chen

**Affiliations:** 1 State Key Laboratory of Resource Insects, Institute of Apicultural Research, Chinese Academy of Agricultural Sciences, Beijing 100093, China; shanjinqiong@caas.cn (J.S.);; 2 Apiculture Technical Guidance Station, Urumqi 831400, China; 3 Faculty of Agricultural Sciences and Food, Ss. Cyril and Methodius University in Skopje, 1000 Skopje, North Macedonia

**Keywords:** honeybees, *Apis mellifera sinisxinyuan*, cold tolerance, transcriptome, differentially expressed genes

## Abstract

**Simple Summary:**

The *Apis mellifera sinisxinyuan* is a kind of western honeybee with strong cold hardiness. However, the cold tolerance mechanism of *A. m. sinisxinyuan* is still not clear. Here, we analyzed the supercooling point and transcriptome data of *A. m. sinisxinyuan* treated with different temperatures and speculated that honeybees increase their energy to resist cold stress through signaling transduction, hormone regulation, oxidative phosphorylation, and other processes to shivering thermogenesis and regulate their carbohydrate and lipid metabolism. This study provides insight into the cold tolerance mechanisms of honeybees.

**Abstract:**

Honeybees are important pollinators worldwide that are closely related to agricultural production and ecological balance. The biological activities and geographical distribution of honeybees are strongly influenced by temperature. However, there is not much research on the cold tolerance of honeybees. The *Apis mellifera sinisxinyuan*, a kind of western honeybee, exhibits strong cold hardiness. Here, we determined that short-term temperature treatment could regulate the honeybee’s cold tolerance ability by measuring the supercooling point of *A. m. sinisxinyuan* treated with different temperatures. Transcriptome data were analyzed between the treated and untreated honeybees. A total of 189 differentially expressed genes were identified. Among them, *Abra*, *Pla1*, *rGC*, *Hr38*, and *Maf* were differentially expressed in all comparisons. GO and KEGG analysis showed that the DEGs were enriched in molecular functions related to disease, signal transduction, metabolism, and the endocrine system’s function. The main components involved were ribosomes, nucleosomes, proteases, and phosphokinases, among others. This study explored the formation and regulation mechanism of cold tolerance in honeybees, not only providing a theoretical basis for cultivating honeybees with excellent traits but also promoting research and practice on insect stress tolerance.

## 1. Introduction

Insects are predominantly distributed in subtropical, temperate, and polar regions of the Earth. Temperature plays a crucial role in regulating their reproduction, distribution, and foraging activities. Cold tolerance refers to the ability of insects to adapt to changes in low-temperature environments, both over extended periods and short durations [1]. This ability is influenced by various factors, including the insect’s developmental stage, growth conditions, genetic factors, the temperature and duration of exposure to cold environments, etc. [2,3]. Over their evolutionary history, insects have developed diverse mechanisms to withstand cold temperatures. Supercooling is considered a common phenomenon observed in insects’ response to cold stress through facilitating intercellular ice growth and preventing the formation of large crystals that may damage tissues [4,5,6]. Insects’ body temperatures decrease with the decrease in environmental temperatures; when the temperatures drop to a certain degree, insects’ body temperatures will suddenly rise and then continue to decline. This temperature is called the supercooling point (SCP), which can change with environmental conditions [7]. Insects also adapt to low-level stress during growth and development through ecological adaptation, coordinated interactions with symbiotic microorganisms, and accumulation of energy substances [8,9,10]. Small molecules, such as mannitol, sorbitol, and trehalose, found in insect hemolymph, along with fatty acids and amino acids, have been confirmed as major cold-resistant substances in insects [11]. Additionally, antifreeze protein (AFP), the circadian clock gene *vrille*, and genes involved in the sugar metabolism pathways, such as trehalase (*Treh*) and trehalose-6-phosphate synthase (*TPS*), have also been confirmed to regulate insect cold tolerance [12,13].

Honeybees, as an important economical insect worldwide, are widely distributed in tropical and temperate regions. Temperature is a key factor influencing the geographical distribution of honeybees and the development of honeybee colonies. The Prevention of honeybee COlony LOSSes (COLOSS) has been monitoring honeybee winter mortality rates in various countries since 2008, finding that the winter loss rates of European honeybees have consistently remained high (ranging from 7.4% to 28.9%) [14,15]. In China, statistical analyses of honeybee winter losses have revealed the winter mortality rate of honeybees exceeds 16% in provinces such as Hainan, Henan, Jiangxi, and Xinjiang [16]. This indicates that the challenge of honeybees’ winter survival has become a major factor limiting the development of the global beekeeping industry. Moreover, the cold tolerance of honeybees will also affect the time they leave the hive in early spring and early morning, which then affects the pollination efficiency and thus crop yields and harvests. Therefore, studying the cold tolerance of honeybees is of paramount importance.

Currently, research on the cold tolerance of honeybees mainly focuses on their physical and chemical properties as well as the accumulation of related substances. Studies have shown that cold stress can significantly affect the levels of fat, glycogen, and polyols of honeybees [17,18]. The expression levels of genes related to the antioxidant system, such as TPS, superoxide dismutase (SOD), and catalase (CAT), were also changed under cold stress [19]. Weighted gene co-expression analysis and transcriptome analysis suggest that genes in the Hippo, Foxo, and MARK signaling pathways might help honeybees withstand cold stress [20,21,22] There is limited research on the identification and functional analysis of genes related to honeybee cold tolerance. It has been shown that the myosin family, the C_2_H_2_ zinc finger transcription factor family, and the AFP significantly influence the cold tolerance of the Chinese honeybee [18,21,23]. However, the understanding of the cold tolerance mechanism of honeybees is still limited. More work is required to understand the molecular regulatory mechanisms of cold tolerance in honeybees.

*Apis mellifera sinisxinyuan* is a subspecies of the Western honeybee, naturally distributed in the temperate regions of the Tian Shan mountains. This honeybee species is characterized by a strong foraging ability, rapid reproduction, a strong tolerance to adverse conditions, and high adaptability, making it excellent for studying bee cold tolerance. In previous research, by comparing the temperate populations of *A. m. sinisxinyuan* and *A. m. mellifera* with the tropical populations of *A. m. scutellata*, it was identified that the fat body and the Hippo signaling pathway might play important roles in the temperature adaptation process of honeybees [20]. This study further explores the genes related to cold tolerance in *A. m. sinisxinyuan*. For this, 20-day-old worker bees were collected in the early overwintering period and were treated at different temperatures (4 °C, 10 °C, and 25 °C). Comparative transcriptome analysis was then conducted between these treated honeybees and untreated worker bees. Combined with supercooling point data, the cold tolerance mechanism associated pathways and genes of the *A. m. sinisxinyuan* were investigated. This study offers a theoretical basis for understanding the mechanisms of cold tolerance in honeybees.

## 2. Materials and Methods

### 2.1. Temperature Treatment of Honeybees

*A. m. sinisxinyuan* colonies were maintained in an apiary located in Yili River Valley (Xinjiang Uygur, China). The overwintering bees bred on 28 August 2023 and emerged on 17 September 2023 in three hives with consistent sizes. Within two days, 600 newly emerged worker bees were marked with paint pens in each hive. After 20 days, 120 marked worker bees were randomly collected from each hive when the honeybees were fully mature and in the forager stage. The honeybees were then divided into four groups (30 bees/group) using beekeeping cages.

According to the hive center temperature of 35 °C, three groups were treated with a low temperature at 25 °C (X25), 10 °C (X10), and 4 °C (X4) in incubators. The duration was 2 h, referring to the temperature treatment experiment of *Drosophila melanogaster* [24]. Then, the supercooling points of 20 honeybees in each group were determined using a programmable controller (1 °C/min) (CryoLogic CL–5500) and the supercooling point tester (SUN-V, Beijing Pengcheng Electronic Technology Co., Ltd., Beijing, China), and the remaining honeybees in each group were used to implement RNA-seq. The group without any treatment was used as the control group (NT).

### 2.2. RNA Extraction, Library Preparation, and Sequencing

RNA was extracted from honeybees using the phenol/chloroform technique. The purity of the RNA was evaluated using a NanoDrop 2000 spectrophotometer (Thermo Fisher Scientific, Waltham, MA, USA) and the concentration was determined using an Agilent 4200 TapeStation (Agilent Technologies Inc., Santa Clara, CA, USA). The integrity of the RNA was assessed via the Agilent RNA Screen Tape Assay. After verifying the quality of the total RNA samples, eukaryotic mRNA was enriched using Oligo (dT) magnetic beads. Fragmentation buffer was introduced to fragment the mRNA. The fragmented mRNA then served as a template for synthesizing the first and second strands of cDNA. The resulting cDNA was purified. The purified double-stranded cDNA underwent end repair, A-tailing, and sequencing adapter ligation. Around 350 bp cDNA fragments were selected and recovered for further use. The cDNA library was amplified via PCR. Quality checks of the constructed library were performed using Qubit 3.0 and Agilent 2100 to ensure they met the required standards. The qualified library was sequenced on a high-throughput platform, NovaSeq S4, using the Illumina sequencing method PE150 [25]. This process safeguards the integrity and quality of RNA, ensuring reliable sequencing results for subsequent analyses.

### 2.3. Processing of Raw Data and Statistical Analysis

The raw sequencing data were stored in FASTQ format. The raw sequencing data were filtered using SOAPnuke (v1.5.6) [26] to obtain clean data (–l 15 -q 0.2 -n 0.05). These Clean Reads, free from adapter contamination, low-quality sequences, or those with an N content exceeding 5%, were used for subsequent analysis. Gene and genome annotation files were acquired from NCBI (version *Apis_mellifera*. Amel_HAv3.1). The clean data were aligned to the reference genome using Bowtie2 (v2.3.4.3, -q --phred64 --sensitive --dpad 0 --gbar 99999999 --mp 1,1 --np 1 --score-min L,0,-0.1 -I 1 -X 1000 --no-mixed --no-discordant -p -k 200) [27] and the high-quality sequences were aligned to the reference genome using HISAT2 [28]. HTSeq was employed to count the reads per gene (parameters: -i gene_id -f bam -s no -a 10 -q), and the FPKM values were calculated to estimate the gene expression levels [29,30].

### 2.4. DEGs’ Functional and Pathway Analysis

Differential gene expression was detected using DESeq2 (v1.4.5) [31], with the following criteria: |Log2FoldChange| ≥ 1 and Q value ≤ 0.05. To further explore the functions of genes associated with phenotypic changes, we conducted Gene Ontology (GO) and Kyoto Encyclopedia of Genes and Genomes (KEGG) enrichment analyses of differentially expressed genes using the hypergeometric test via the Phyper function of R (https://stat.ethz.ch/R-manual/R-devel/library/stats/html/Hypergeometric.html) to calculate the *p*-value on 1 February 2024. Then, we performed multiple checks on the *p*-value to verify its positive value; the calibration software package was qvalue (https://bioconductor.org/packages/release/bioc/html/qvalue.html, accessed on 1 February 2024). The threshold for significance was set at a Q-value ≤ 0.05, with enrichment in candidate genes considered significant when this condition was met.

### 2.5. RT-qPCR Validation of DEGs

The PrimeScript™ RT Reagent Kit with gDNA Eraser (Perfect Real Time) (TaKaRa Biotechnology, Dalian, China) was used to synthesize first-strand cDNA according to the manufacturer’s instructions. The relative expressions of gene transcripts were determined using quantitative real-time PCR (qPCR), and the internal control was set using the *β*-actin gene [32]. The qPCR was conducted with the LineGene 9600 plus quantitative fluorescence PCR System. The SYBR Premix Ex Taq kit (TaKaRa Biotechnology) was used according to the standard PCR protocol, and the program was set as follows: 95 °C for 30 s, followed by 40 cycles at 95 °C for 5 s and 60 °C for 34 s. One dissociation step cycle of 95 °C for 15 s, 60 °C for 1 min, 95 °C for 30 s, and 60 °C for 15 s was set to ensure the product specificity after amplification. The 2^–ΔΔCt^ method was used to calculate the relative expression of the gene transcripts [33]. All the primers for the qPCR are provided in the Supplementary Data (Table S1).

### 2.6. Statistical Analysis

The statistical analyses between groups were executed using Student’s *t*-test. ANOVA, followed by the Tukey HSD test, was carried out to determine the statistical differences of multiple groups. The mean ± standard deviation (SD) was used to present the descriptive statistics. All pictures were structured using Prism 8.1 (GraphPad Software Inc., La Jolla, CA, USA), and all data were analyzed using SPSS v23 (SPSS software Inc., Chicago, IL, USA). *p*-values < 0.05 were considered to be significant.

## 3. Results

### 3.1. Supercooling Point in Different Groups of A. m. sinisxinyuan

**Figure 1:**
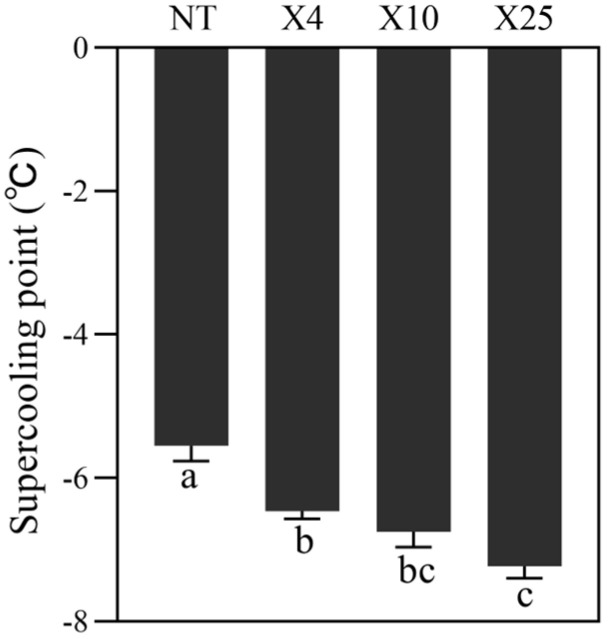
Supercooling point of honeybees in different groups. Different letters mean significant differences among groups (one-way ANOVA followed by Tukey’s multiple comparison test, *p* < 0.05).

The supercooling point (SCP) of different groups was determined after treating *A. m. sinisxinyuan* with different temperatures. The results showed that the SCP of the X25 (−7.22), X10 (−6.74), and X4 (−6.46) groups was significantly lower than the NT (−5.44) group (*p* < 0.05). The X25 had the highest SCP among these groups, significantly higher than the X4 and NT groups, respectively (Figure 1) (*p* < 0.05). It could be inferred that short-term temperature treatment could stimulate *A. m. sinisxinyuan* cold tolerance.

### 3.2. RNA Sequencing Data Quality Assessment

We obtained three samples in the NT group, two samples in the X4 group (one sample was excluded due to low sample correlation), three samples in the X10 group, and three samples in the X25 group. Each sample generated 47.19 (M) of raw reads. After quality control, the number of clean reads ranged from 44.63 to 45.46 million (M), with Q20 values greater than 97% and Q30 values greater than 93%. When the transcriptome sequences were aligned with the NCBI species genome (version *Apis_mellifera*. Amel_HAv3.1), more than 77% of the sequences were uniquely mapped. The high-quality RNA-seq data can be utilized for further transcriptomic analysis (Table S2).

### 3.3. Differential Gene Expression Analysis

**Figure 2:**
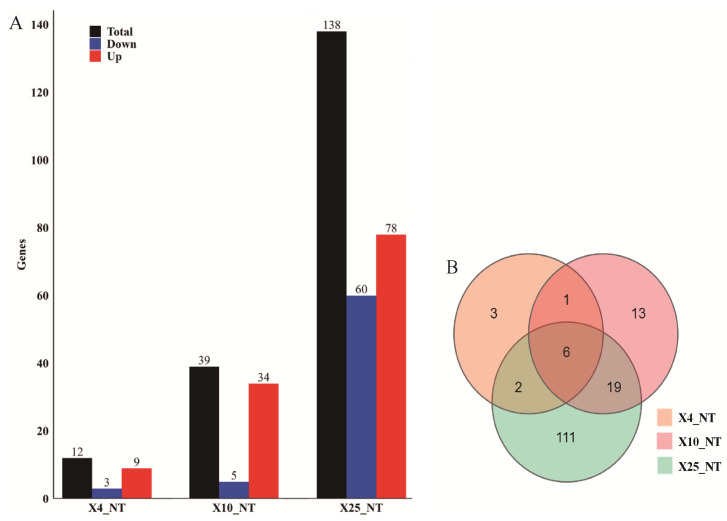
DEG analysis chart. (**A**) Bar chart of the number of differential genes in multiple groups: Red represents the upregulated genes, blue represents the downregulated genes, and black represents all the differential genes (*p* < 0.05). The *x*-axis indicates the number of genes. (**B**) Venn diagram of the DEGs between different groups.

In the comparisons of X4, X10, and X25 against NT, there were 12, 39, and 138 differentially expressed genes identified, respectively (Figure 2A). Specifically, in the X4_NT comparison, nine genes were upregulated, and three were downregulated. In the X10_NT comparison, thirty-four genes were upregulated, and five were downregulated. The X25_NT comparison showed the highest number of differentially expressed genes, with 78 upregulated and 60 downregulated genes (Figure 2A). A Venn diagram analysis of all differentially expressed genes across the three comparisons revealed six common differentially expressed genes. Among these, five were upregulated: actin-binding Rho-activating protein (*Abra*, LOC410370), phospholipase A1 (*Pla1*, LOC410884), uncharacterized gene (*UC*, LOC411290), receptor-type guanylate cyclase gcy-4 (*rGC*, LOC413596), and Hormone receptor-like in 38 (*Hr38*, LOC551232). One gene, transcription factor Maf (*Maf*, LOC724861), was downregulated (Figure 2B). It is hypothesized that these genes play important regulatory roles in cold tolerance in honeybees and may be considered candidate genes influencing honeybee cold tolerance.

### 3.4. Functional Pathway Enrichment Analysis

**Figure 3:**
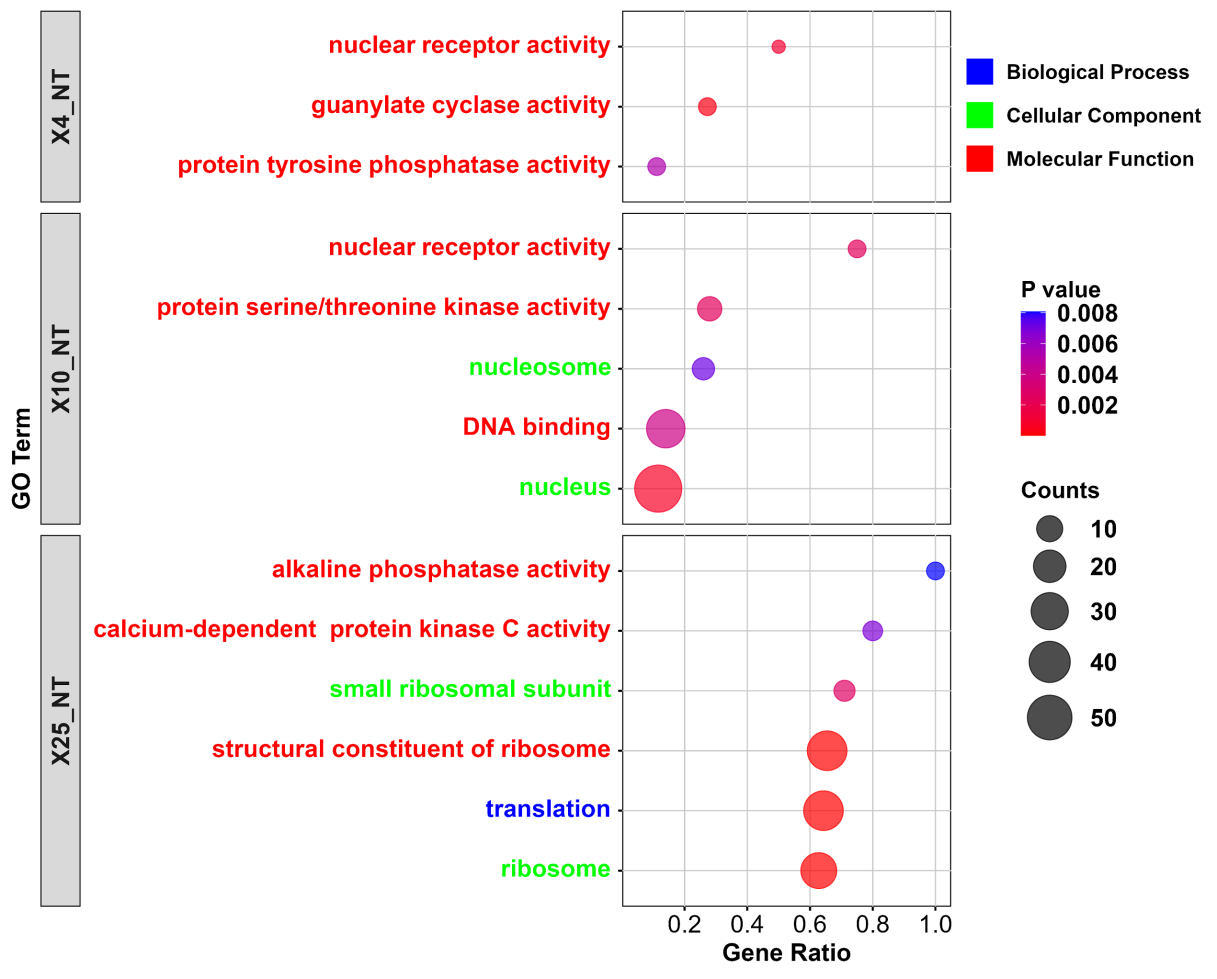
GO enrichment analysis bubble plot: the *y*-axis represents the GO terms, the *x*-axis represents the ratio of enriched genes in each term, the bubble size indicates the number of enriched genes, and the color represents the *p*-value, with red indicating higher significance.

The identified differentially expressed genes (DEGs) were subjected to GO functional annotation and KEGG pathway enrichment analysis to elucidate differences at the gene level between samples. This approach aids in identifying target genes and key pathways. GO annotation statistics were generated for the DEGs in both the treatment and control groups, and a bar chart was produced to represent the GO annotations (Figure 3). The functional annotations of the DEGs were categorized into three major groups: molecular function, biological process, and cellular components. In this study, GO enrichment of DEGs was primarily focused on molecular function. The comparisons of X4_NT, X10_NT, and X25_NT yielded three, five, and six significantly enriched GO terms, respectively (*p* < 0.05). Among these, “nuclear receptor activity” appeared twice, and enzyme activity-related GO terms were present in each comparison. These functional terms mainly involved ribosomal components, nucleosomes, nuclear receptors, and various enzymes such as proteases and phosphokinases (Figure 3). These components may play crucial roles in cold tolerance and cold stress-related functions in honeybees.

**Figure 4:**
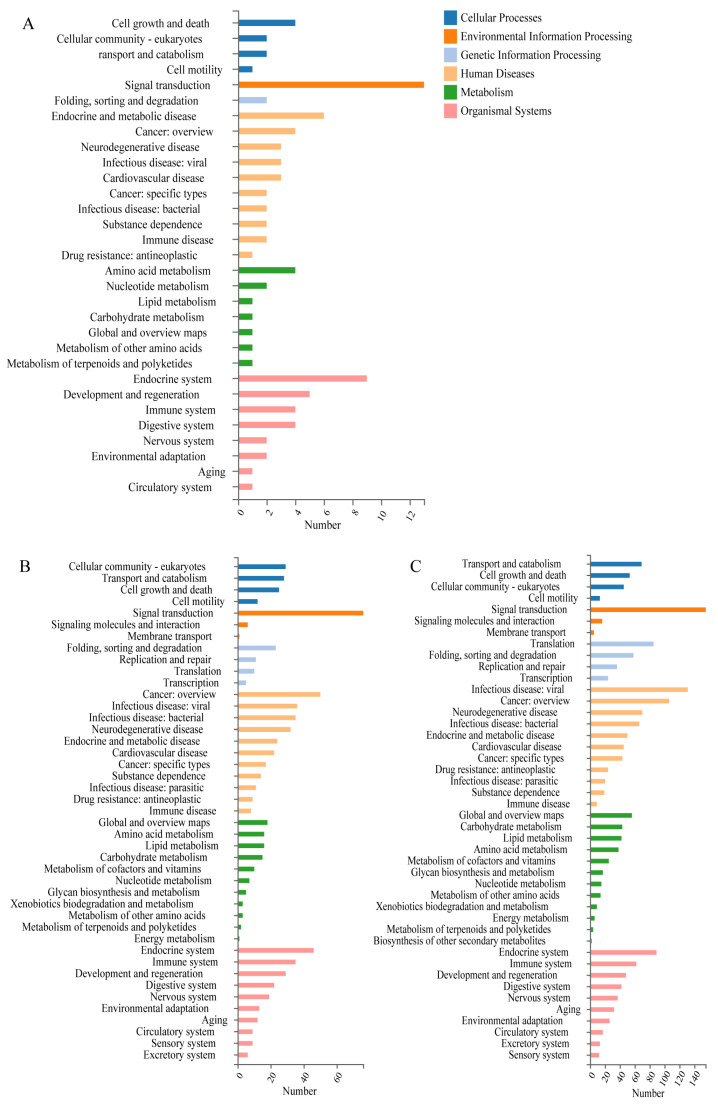
Pathways annotated by DEGs. (**A**) DEGs in X4_NT. (**B**) DEGs in X10_NT. (**C**) DEGs in X25_NT.

Additionally, KEGG pathway analysis indicated that the DEGs in the X4_NT comparison were associated with 119 pathways, with the most enriched categories being human diseases and organismal systems. Under the secondary classifications, pathways related to signal transduction, endocrine and metabolic diseases, and the endocrine system were predominant (Figure 4A). The X10_NT comparison revealed that the DEGs were associated with 299 pathways, with the most enriched categories being metabolism, human diseases, and organismal systems. Secondary classifications showed a high prevalence of pathways related to signal transduction, cancer: overview, and the endocrine system (Figure 4B). The X25_NT comparison had the highest number of associated pathways, with 334 pathways identified. The most enriched primary categories were human diseases and organismal systems, with secondary classifications highlighting signal transduction, infectious disease: viral, and the endocrine system (Figure 4C). Overall, the enriched pathways across the three comparisons were largely consistent, focusing on functions related to disease, signal transduction, metabolism, and the endocrine system. It is hypothesized that cold stimuli in honeybees may trigger changes in metabolism and endocrine function, involving signal transduction processes similar to those observed in the body’s response to disease.

### 3.5. Validation of RNA-seq via qRT-PCR

**Figure 5:**
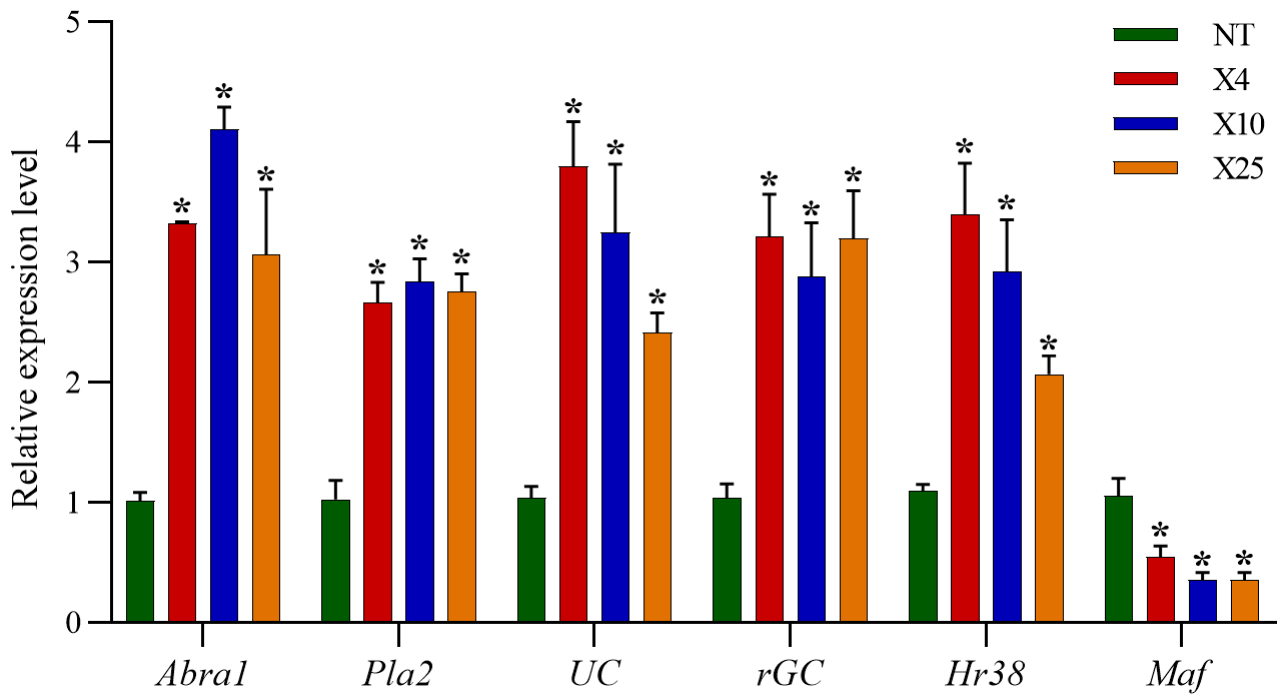
Relative expression levels of DEGs in different groups of honeybees according to RT-qPCR. Bars with an asterisk mean significant differences between NT and others (Student’s *t*-test, *p* < 0.05).

In order to validate the expression profiles from the RNA-seq, we carried out qRT-PCR on the cold tolerance-related DEGs (*Abra1*, *Pla1*, *UC*, *rGC*, *Hr38* and *Maf*). The results showed that the expression patterns of six genes from the qRT-PCR (Figure 5) were consistent with those from the RNA-seq (Table 1). However, the fold change of expression patterns between qRT-PCR and RNA-seq still deviated slightly, which might be due to methodological differences.

## 4. Discussion

Insects have physiological and behavioral changes in cold environments to resist cold environmental conditions [34,35]. Adjusting the supercooling point was often considered an active way to adapt to the environment [7,36]. In honeybees, the SCP was highly consistent with the trend of changes in the external environmental temperature; the supercooling ability increased when the external temperature declined, which helped bees to avoid freezing damage and resist the threat of winter temperature [17]. Here, we determined the SCP of *A. m. sinisxinyuan* after different temperature treatments; the results showed that short-term temperature treatments could increase the supercooling ability. This is consistent with previous research on *Schizaphis graminum*, where first instar nymphs of the greenbug aphid exhibited stronger cold tolerance compared to wingless adults, with significantly lower supercooling points [37]. This suggests that a lower supercooling point indicates a stronger ability to tolerate cold stress. 

Comparative transcriptome analysis of *A. m. sinisxinyuan* treated with different temperature was executed to further explore the mechanism of cold tolerance. There were a total of 189 differentially expressed genes identified compared with the untreated honeybees. The higher temperature correlated with an increased number of differentially expressed genes, with a greater number of upregulated genes compared to downregulated ones, which is consistent with the SCP results. The reason might be that the excessively low temperature actually inhibits the metabolism of honeybees, and slightly lower temperatures are more likely to induce gene changes related to cold tolerance. Combining GO and KEGG enrichment analyses, we speculate that bees increase their energy to resist cold stress through shivering thermogenesis and by regulating their carbohydrate and lipid metabolism. This process likely involves signaling transduction, hormone regulation, oxidative phosphorylation, and other processes, ultimately affecting the metabolism, endocrine system, hemolymph circulation, and immune system of honeybees. Additionally, six differentially expressed genes (DEGs) were identified in all comparisons: actin-binding Rho-activating protein (*Abra*), phospholipase A1 (*Pla1*), uncharacterized gene (*UC*), receptor-type guanylate cyclase gcy-4 (*rGC*), Hormone receptor-like in 38 (*Hr38*), and transcription factor Maf (*Maf*). Except for the *UC*, the remaining genes have been previously researched.

*Abra*, also known as the striated muscle activator of the Rho signaling (STARS) protein, is highly enriched in mammalian cardiac, skeletal, and smooth muscles, playing a significant role in intracellular signaling that influences various cellular processes within muscle tissues [38,39]. The contraction and relaxation of muscles related to blood circulation, such as smooth and cardiac muscles, are influenced by the animal’s response to cold and heat. The unique muscle structure of honeybees suggests that their muscle movements may also be regulated under temperature stress. Worker bees’ flight muscles are crucial for maintaining the colony’s constant temperature. To cool down, workers fan hot air out of the hive, creating airflow that brings in cooler air [40,41]. To warm up, workers generate heat through shivering in their indirect flight muscles, a process known as shivering thermogenesis [42,43]. In previous studies, *flightless I*, related to the tissue structure of indirect flight muscles, exhibited a significant positive selection signal in temperate populations [20]. Here, we speculate that *Abra* may regulate honeybees’ flight muscle activity, enabling heat generation through muscle shivering in cold environments.

*Maf* was first discovered as an oncogene family in avian tumor viruses and was later shown to act as a transcription factor [44]. Maf transcription factors play active roles in the development, differentiation, and establishment of specific functions in various organs, tissues, and cells, such as the pancreas [45]. *Maf* participates in insulin gene transcription and can regulate the body’s glucose/energy balance [46]. When insects are exposed to cold stress, sugars often act as cryoprotectants, protecting partially frozen tissues, and may contribute to reducing the supercooling point to prevent bodily fluids from freezing [47]. Freeze-tolerant insects have high sugar levels, such as trehalose, which regulates life activities by modulating hemolymph trehalose concentration under environmental stress [48,49]. Although the specific role of *Maf* in insects is not yet clearly established, if its function is similar to its homologs in mammals, it may regulate sugar metabolism in bees and other insects to cope with cold stress. Aside from hemolymph, the fat body is also an essential energy storage organ in bees. The fat body can utilize stored energy substance to produce glycerol, sorbitol, and other compounds to lower the SCP and enhance the cold tolerance [50]. *Pla1* is responsible for generating energy by hydrolyzing lipids, at the sn-1 position of phospholipid molecules, into lysophospholipids (LPLs). LPLs have various functions in cell signaling and membrane structure regulation, activating signaling pathways by binding to specific receptors and regulating cell growth, differentiation, survival, and other processes [51,52]. Lysophosphatidic acid (LPA), a specific form of lysophospholipid, is involved in cellular processes, including cell proliferation, platelet aggregation, smooth muscle contraction, and neurite retraction [45,53]. LPA can also induce smooth muscle cell transformation. Several candidate genes play a role in regulating the function of smooth muscle and blood vessels. The movement of vascular smooth muscle is highly influenced by the environmental temperature. During cold stress, vascular smooth muscle contracts, limiting blood flow to the skin surface, reducing heat loss, and helping to maintain the core body temperature. This response is closely tied to the cold tolerance of honeybees [54]. The orientation of several candidate genes toward smooth muscle and vascular function suggests that these functions are significantly related to bee cold tolerance. Guanylate cyclase C (GC-C) activates diet-induced thermogenesis in brown adipose tissue (BAT) and plays a crucial role in regulating glucose homeostasis and insulin regulation [55]. Cold exposure activates brown adipose tissue, secreting signaling lipids to improve metabolism, increasing LPL levels, and secreting oxylipin 12-hydroxyeicosapentaenoic acid (12-HEPE) to enhance glucose uptake in fat tissue and skeletal muscle [56,57]. Although insects lack BAT, they may possess similar guanylate cyclase signaling mechanisms performing analogous functions.

*Hr38* is an immediate early gene (IEG) that is rapidly and transiently expressed following neuronal stimulation, serving as a tool to identify brain regions involved in molecular processes related to complex behavior and neural plasticity [58]. *Hr38* is enriched in mushroom body neurons and transduces ecdysone signaling [59]. Cold stress inhibits ecdysone synthesis, and low temperatures can block ecdysone signaling, thereby regulating bee development and reproduction. Ecdysone regulates both ovarian development and the mating behavior of queen bees while also maintaining the colony’s social structure by inhibiting the reproductive capabilities of worker bees. This complex mechanism allows honeybees to reproduce efficiently within their highly social colonies, ensuring the colony’s long-term survival and success [60]. The mushroom body (MB) is a higher center in the insect brain involved in learning, memory, and multimodal sensory integration [61]. The dendrites of Kenyon cells form the cup-shaped calyx of the mushroom body, an important area for sensory input [62]. Temperatures also affect the development of the mushroom body [60]. Given that cold stress impacts the development of the mushroom body, a regulatory area for various sensory and hormonal processes in bees, *Hr38*, as an IEG significantly enriched in the mushroom body, may play a crucial role in responding to temperature changes. Additionally, *Hr38* and Kenyon cell expression is upregulated by foraging flight in worker bees (with higher expression levels in foragers compared to nurse bees) [63], which may influence the strength of bee flight muscles and, consequently, their cold tolerance.

## 5. Conclusions

In conclusion, this study showed that the overall transcriptome dynamics of *A. m. sinisxinyuan* was altered across temperature treatments, and the genes related to metabolism of carbohydrate and lipids regulated by insulin and shivering thermogenesis are induced to facilitate the cold tolerance of honeybees. This study preliminary explored the cold tolerance mechanism of honeybees. Future studies can focus on the verification and analyses of the function of DEGs identified here and clarify the cold tolerance mechanism of honeybees, providing a theoretical basis for breeding honeybees with cold tolerance. Our results provide insight into the cold tolerance mechanisms of honeybees and offer a basis for exploring insect stress tolerance.

## Figures and Tables

**Table 1:** Comparison of expression levels of DEGs in different groups in transcriptomic data.

Gene Name	X4_NT		X10_NT		X25_NT	
	Log2^Fold change^	Q-Value	Log2^Fold change^	Q-Value	Log2^Fold change^	Q-Value
*Abra1*	1.12	2.38 × 10^−3^	1.58	1.47 × 10^−20^	1.45	4.41 × 10^−9^
*Pla1*	1.34	4.31 × 10^−3^	1.27	2.16 × 10^−04^	1.32	6.25 × 10^−6^
*UC*	1.37	2.77 × 10^−16^	1.15	8.19 × 10^−33^	1.34	4.20 × 10^−8^
*rGC*	1.34	7.48 × 10^−9^	1.65	6.15 × 10^−25^	1.99	1.05 × 10^−26^
*Hr38*	2.40	3.85 × 10^−47^	1.99	1.53 × 10^−26^	2.40	3.65 × 10^−29^
*Maf*	−1.43	1.68 × 10^−6^	−1.44	2.51 × 10^−16^	−1.54	1.07 × 10^−11^

## Data Availability

The data supporting this article have been included as part of Table S3 in the Supplementary Materials.

## Supplementary Materials

The following supporting information can be downloaded at https://www.mdpi.com/article/10.3390/insects15100790/s1, Table S1: Primers for RT-qPCR validation in this study; Table S2: Summary of RNA-seq data for data availability statements; Table S3: Summary of RNA-seq data for data availability statements (DAS).
