# Supplementary material for: Comparative Transcriptome Analysis of Cold Tolerance Mechanism in Honeybees (Apis mellifera sinisxinyuan)
Source: Insects. 2024 Oct 11;15(10):790. doi: 10.3390/insects15100790 (PMC11508713; doi:10.3390/insects15100790)
Supplement: Supplementary file 1 [file insects-15-00790-s001.zip › insects-3193751-supplementary.pdf]

Supplementary Materials:

Table S1 Primer information of DEGs for RT-qPCR

Gene ID	Primer name	Sequence (5'-3')	Length (base pair)
410370	Abra1-F	TGTGTAGGACTTTTGCTGAGAG	143
	Abra1-R	GCACATCATCGTCTCGTCTC	
410884	Pla2-F	TTTACACGAATGACAGGCCGA	87
	Pla2-R	GTGTCGTCACCACCTGCAT	
411290	UC-F	AGAACTCCAACCACACCAGT	73
	UC-R	CTGCTGCCAACTAAGACCTTC	
413596	rGC-F	ACAAGACGGTGGTGATCAGT	121
	rGC-R	GCTTGTTCTCGCCGATAGTC	
551232	Hr38-F	GCCTGACTCGCTCAGTTCAA	109
	Hr38-R	AAGAACCTGAAGCTCCTGCC	
724861	Maf-F	CCGCGGGAAGAGGTCGTA	83
	Maf-R	TCGTTGTTGAAGTCGCTTGC	

Table S2 Quality assessment of transcriptome data

Sample	Total Raw Reads (M)	Total Clean Reads (M)	Q20 (%)	Q30 (%)	Clean Reads Ratio (%)	Total Mapping (%)	Uniquely Mapping(%)
NT-1	47.19	44.63	97.92	93.59	94.58	97.32	95.73
NT-2	47.19	45.27	97.97	93.77	95.93	91.62	90.09
NT-3	47.19	45.19	97.76	93.11	95.76	92.73	91.03
X4-1	47.19	45.11	98.00	93.82	95.59	88.13	86.60
X4-2	47.19	45.14	98.44	94.33	95.66	97.66	96.12
X10-1	47.19	45.28	97.83	93.27	95.95	78.46	77.17
X10-2	47.19	45.40	97.97	93.72	96.21	87.95	86.45
X10-3	47.19	45.14	97.93	93.65	95.66	91.58	90.15
X25-1	47.19	45.46	98.45	94.38	96.33	92.02	90.58
X25-2	47.19	45.24	98.43	94.32	95.87	97.58	96.02
X25-3	47.19	45.31	98.38	94.16	96.02	97.10	95.44

Data availability statements (DAS):

Table S3: Summary of RNA-seq data for data availability statements.

Gene ID	Log^2 (X4_X0)	Q-value (X4_X0)	Log^2 (X10_X0)	Q-value (X10_X0)	Log^2 (X25_X0)	Q-value (X25_X0)
100576198	0.630	0.866	0.981	0.027	1.200	0.014
100576223	-0.827	0.438	-0.558	0.138	-1.227	0.001
100576233	-0.935	0.250	-0.630	0.094	-1.175	0.001
100576265	-0.256	0.981	-0.237	0.804	-1.785	0.045
100576271	-0.376	0.552	-0.528	0.001	-1.077	0.000
100576395	1.356	0.317	1.606	0.000	2.316	0.000
100576935	0.657	0.292	0.917	0.001	1.173	0.000
100577065	-2.575	0.451	-1.851	0.082	-3.626	0.013
100577091	0.145	0.986	1.073	0.045	1.149	0.091
100577118	0.774	0.514	0.863	0.005	1.605	0.000
100577142	0.412	0.010	0.858	0.000	1.122	0.000
100577222	-1.509	0.322	-0.483	0.480	-2.069	0.001
100577224	1.059	0.005	0.908	0.000	0.731	0.000
100577331	1.313	0.746	1.662	0.080	2.778	0.041
100577358	-0.930	0.091	-0.437	0.161	-1.241	0.000
100577436	-2.433	0.140	-2.100	0.000	-2.788	0.001
100577614	-0.489	0.425	-0.701	0.000	-1.307	0.000
100577777	-1.255	0.013	-0.399	0.316	-0.572	0.176
100577825	-0.603	0.869	-0.211	0.756	-1.762	0.002
100577936	0.466	0.826	1.156	0.000	0.786	0.018
100577939	-1.048	0.746	-0.111	0.912	-1.667	0.028
100578112	0.860	0.360	0.596	0.043	1.222	0.000
100578309	-0.697	0.293	-0.809	0.000	-1.274	0.000
100578437	0.880	0.000	1.482	0.000	1.510	0.000
100578547	-1.436	0.451	-1.258	0.022	-1.843	0.005
100578739	0.395	0.894	0.844	0.026	1.174	0.000
100578744	-0.769	0.639	-0.184	0.757	-1.069	0.019
100578814	-0.416	0.940	1.198	0.103	1.105	0.044
100578881	0.229	0.980	0.571	0.299	1.010	0.029
100578955	0.859	0.001	0.811	0.000	1.069	0.000
102653673	-0.764	0.558	0.501	0.172	-1.088	0.015
102653784	-1.215	0.383	-0.358	0.561	-1.046	0.048
102653798	-0.715	0.631	-0.753	0.053	-1.317	0.001
102653979	-1.220	0.850	-1.083	0.410	-3.268	0.018
102654066	-0.267	0.952	-0.120	0.846	-1.007	0.013
102654315	-0.258	0.971	-0.145	0.857	-1.544	0.017
102654366	0.513	0.963	0.598	0.608	1.445	0.050
102654530	-0.149	0.982	-0.227	0.742	-1.191	0.047
102654579	-0.319	0.959	-0.780	0.108	-1.547	0.003

102654641	-1.154	0.705	-0.735	0.330	-1.569	0.030
102654726	0.745	0.898	0.890	0.231	1.731	0.002
102654860	0.147	0.987	1.259	0.131	1.795	0.006
102655138	-1.143	0.746	-0.871	0.259	-1.563	0.049
102655332	0.788	0.002	0.620	0.000	1.005	0.000
102655538	-0.291	0.960	-0.378	0.565	-1.152	0.034
102655710	-0.304	0.957	-0.680	0.198	-1.381	0.010
102655788	0.854	0.051	0.744	0.002	1.272	0.000
102656221	0.787	0.920	1.512	0.038	0.767	0.504
102656464	-0.951	0.454	-0.155	0.818	-1.195	0.011
102656505	0.403	0.974	1.583	0.005	0.645	0.513
102656664	-2.982	0.002	-0.979	0.052	-2.250	0.000
102656830	2.372	0.310	1.544	0.135	2.418	0.009
107963975	-0.102	0.986	1.532	0.030	0.538	0.534
107964039	-0.501	0.893	-0.432	0.429	-1.068	0.040
107964284	0.820	0.790	1.289	0.028	1.392	0.007
107964765	0.407	0.956	1.120	0.025	0.531	0.434
107964774	-0.732	0.773	-0.639	0.221	-1.203	0.031
107964975	-0.683	0.971	-0.612	0.750	-4.127	0.031
107964994	-0.314	0.957	-0.590	0.273	-1.591	0.005
107965055	-1.198	0.642	-0.326	0.723	-1.578	0.039
107965485	-0.924	0.536	-0.929	0.070	-1.246	0.013
107966050	-0.518	0.913	-0.480	0.502	-1.671	0.007
113218569	1.009	0.220	0.954	0.005	1.065	0.004
113218672	-0.155	0.985	-0.425	0.553	-1.537	0.022
113218760	- .0	1.000	20.996	0.000	5.319	0.120
113218848	-0.126	0.991	0.933	0.404	2.353	0.001
113218907	1.647	0.044	1.606	0.000	0.965	0.011
113219002	-1.252	0.844	-0.366	0.758	1.260	0.049
113219014	-0.914	0.551	-0.120	0.866	-1.261	0.011
113219092	1.490	0.268	1.320	0.010	1.153	0.120
113219226	1.076	0.633	1.988	0.000	1.516	0.009
113219265	0.069	0.991	1.025	0.210	2.018	0.002
113219380	1.077	0.000	0.274	0.450	-0.444	0.015
408358	0.663	0.062	0.524	0.002	1.051	0.000
408394	-0.551	0.571	-0.624	0.031	-1.081	0.000
408406	0.335	0.844	1.246	0.000	2.060	0.000
408586	0.530	0.826	0.828	0.011	1.033	0.003
408643	0.967	0.009	0.941	0.000	1.111	0.000
408844	0.523	0.002	1.040	0.000	0.849	0.000
408928	1.332	0.493	1.255	0.011	1.530	0.008
408930	0.619	0.462	0.768	0.001	1.304	0.000
409159	0.924	0.250	1.192	0.000	1.479	0.000
409638	0.731	0.751	0.847	0.073	1.344	0.002

410006	-0.918	0.233	-0.921	0.002	-1.394	0.000
410038	-0.843	0.339	-0.711	0.019	-1.398	0.000
410175	0.887	0.360	0.795	0.018	1.474	0.000
410201	0.571	0.007	0.691	0.000	1.065	0.000
410326	1.055	0.407	1.600	0.000	1.115	0.017
410370	1.116	0.002	1.583	0.000	1.445	0.000
410479	-0.110	0.981	0.300	0.625	1.007	0.007
410509	-0.461	0.746	-0.612	0.050	-1.400	0.000
410520	0.850	0.779	0.862	0.139	1.296	0.029
410614	0.912	0.000	1.288	0.000	1.564	0.000
410620	1.362	0.395	1.138	0.022	1.928	0.000
410626	0.710	0.858	0.648	0.261	1.597	0.002
410638	0.577	0.004	0.770	0.000	1.164	0.000
410732	0.571	0.075	1.176	0.000	1.175	0.000
410746	-0.750	0.632	-0.167	0.842	-1.139	0.016
410801	-0.204	0.974	1.048	0.006	0.507	0.230
410884	1.338	0.004	1.267	0.000	1.317	0.000
411011	0.709	0.132	0.747	0.000	1.021	0.000
411207	0.492	0.508	0.895	0.000	1.023	0.000
411290	1.371	0.000	1.151	0.000	1.343	0.000
411414	-0.956	0.510	-0.753	0.093	-1.528	0.001
411494	0.799	0.536	1.042	0.001	0.961	0.013
411552	1.308	0.349	1.362	0.003	2.165	0.000
412057	-0.956	0.493	-0.408	0.443	-1.584	0.001
412197	0.636	0.091	0.701	0.000	1.125	0.000
412230	-0.347	0.814	-0.486	0.063	-1.023	0.000
412540	0.387	0.407	0.641	0.000	1.137	0.000
412883	0.479	0.842	0.737	0.035	1.284	0.000
413054	0.356	0.124	0.453	0.000	1.003	0.000
413113	0.742	0.148	0.762	0.001	1.062	0.000
413596	1.340	0.000	1.654	0.000	1.990	0.000
413789	0.381	0.814	0.457	0.145	1.016	0.000
413892	0.673	0.305	0.459	0.111	1.167	0.000
413934	0.682	0.199	0.591	0.004	1.149	0.000
550918	0.360	0.963	0.502	0.435	1.348	0.021
550965	0.731	0.567	1.200	0.000	1.720	0.001
551044	0.301	0.933	0.679	0.059	1.221	0.001
551232	2.400	0.000	1.992	0.000	2.403	0.000
552030	0.790	0.041	0.732	0.000	1.074	0.000
552096	1.235	0.536	1.420	0.009	2.099	0.000
552186	-0.873	0.464	-0.677	0.077	-1.422	0.000
552281	0.581	0.844	0.832	0.116	1.268	0.016
552297	0.220	0.944	0.688	0.003	1.148	0.000
552357	-0.812	0.746	-0.597	0.294	-1.837	0.000

552592	0.623	0.001	0.646	0.000	1.215	0.000
724274	0.869	0.096	1.019	0.000	1.604	0.000
724367	1.583	0.065	1.794	0.000	2.425	0.000
724395	-0.722	0.856	-0.706	0.226	-2.200	0.000
724488	0.543	0.514	0.673	0.008	1.101	0.000
724571	-0.516	0.897	-0.953	0.033	-1.294	0.010
724861	-1.432	0.000	-1.442	0.000	-1.538	0.000
724867	0.852	0.143	0.861	0.006	1.462	0.000
724921	-0.836	0.007	-1.015	0.000	-0.411	0.090
724993	0.164	0.963	0.745	0.001	1.083	0.000
725003	-0.194	0.980	-0.421	0.507	-1.112	0.026
725247	0.491	0.850	0.637	0.064	1.429	0.000
725407	-1.125	0.309	-0.755	0.099	-1.032	0.026
725438	-0.673	0.751	-0.573	0.318	-1.591	0.001
725668	0.632	0.708	0.618	0.126	1.210	0.001
725671	0.833	0.581	0.980	0.021	1.282	0.003
725784	-0.960	0.037	-0.946	0.000	-1.248	0.000
725827	0.540	0.235	0.799	0.000	1.212	0.000
725890	-0.787	0.418	-0.477	0.193	-1.438	0.000
726074	-0.743	0.486	-0.651	0.045	-1.271	0.001
726200	-0.851	0.073	-1.007	0.000	-0.991	0.001
726367	-1.008	0.779	-0.413	0.630	-1.876	0.014
726451	-0.593	0.842	-0.617	0.170	-1.911	0.000
726478	-0.914	0.004	-0.762	0.000	-1.091	0.000
726803	0.959	0.028	1.386	0.000	1.773	0.000
727091	-0.582	0.570	-0.353	0.291	-1.040	0.001
727423	1.030	0.000	0.881	0.000	1.215	0.000
727486	0.862	0.002	0.651	0.000	1.005	0.000
